# Managing a Misdiagnosed Case of Nevus Sebaceous

**DOI:** 10.7759/cureus.62184

**Published:** 2024-06-11

**Authors:** Kshitiz Lakhey, Namratha Puttur, Rohan Manoj, Priya Garg, Nishtha Malik

**Affiliations:** 1 Dermatology, Venereology, and Leprosy, Dr. D. Y. Patil Medical College, Hospital & Research Centre, Dr. D. Y. Patil Vidyapeeth (Deemed to be University), Pune, IND

**Keywords:** dermoscopy, histopathology examination, co2 laser, erbium:yag laser, full thickness excision, nevus sebaceous of jadassohn, nevus sebaceous

## Abstract

A male patient in his early 20s presented to our outpatient clinic, having previously been misdiagnosed and unsuccessfully treated as a case of viral warts. Dermoscopic and histopathological evaluations revealed characteristic features of the nevus sebaceous. The lesion was eventually treated with an erbium-doped yttrium aluminum garnet (Er:YAG) laser after the patient declined surgical excision. Nevus sebaceous often presents with verrucous surfaces that make misdiagnosis common. A correct diagnosis is crucial due to potential neoplastic transformations. Histopathological analysis is essential for both the confirmation of disease and the exclusion of malignancy. Full-thickness surgical excision remains the preferred treatment.

## Introduction

Verrucous lesions are defined as those pertaining to or marked by a wart-like growth pattern. Cutaneous warts are, therefore, one of the most diagnosed among such lesions, as they account for approximately 10%-20% of all visits to dermatologists [1]. However, such an appearance is not exclusive to warts. A multitude of lesions may present with a verrucous surface, such as mycobacterial infections [2,3], genodermatosis [4], and vesicobullous disorders [5]. Similarly, autoimmune disorders [6] and neoplasms, both benign and malignant, may mimic warts [7-9]. Nevus sebaceous is one such lesion that may be misdiagnosed based on its appearance.

## Case presentation

A male patient in his early 20s presented to the dermatology outpatient clinic with a lesion on his scalp that he initially noticed seven years ago. He had previously consulted multiple general practitioners and had been treated as a case of viral wart and administered five doses of measles-mumps-rubella (MMR) injection into the lesion, which did not result in a reduction in the size of the lesion.

**Figure 1:**
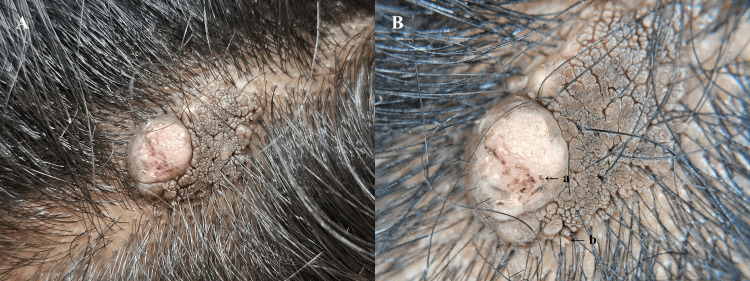
A: Gross examination of the lesion showing a verrucous surface and solitary nodule. B: Appearance of the lesion under polarized dermoscopy (DermLite DL5, Aliso Viejo, California) A: Polymorphic vessels; B: exophytic papules

On examination, a skin-colored, elevated plaque with a verrucous surface was observed, which was soft on palpation. A reduction in the number of terminal hairs arising from the lesion was observed. A single, firm nodule was also noted on one side of the lesion (Figure 1A). Dermoscopy revealed brown globules aggregated in clusters in a cerebriform pattern, along with a few exophytic papillary projections, polymorphic vessels, and white-yellow scales (Figure 1B).

**Figure 2:**
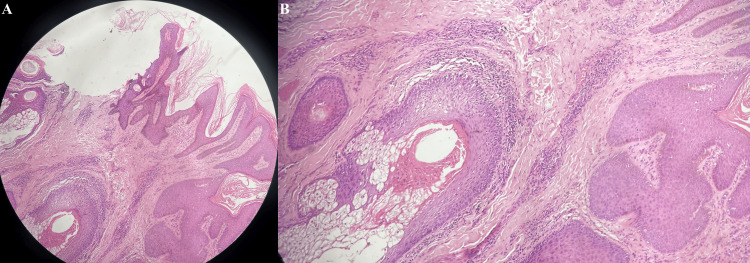
A: Hematoxylin and eosin (H&E) section showing acanthosis, papillomatosis, and abnormal pilosebaceous units (10x). B: Higher magnification showing the abnormal epidermis and pilosebaceous units (40x)

The histopathological examination performed following a 4 mm punch biopsy from the lesion showed acanthosis and papillomatosis of the epidermis with sebaceous glands located in the superficial dermis, occasionally opening directly onto the epidermal surface. Furthermore, a chronic inflammatory infiltrate comprising of lymphocytes was observed in the sub-epidermal region. There was no evidence of atypia, granulomas, or malignancies (Figure 2).

A diagnosis of nevus sebaceous was made based on these clinical, dermoscopic, and histopathological findings. The patient was initially recommended a surgical excision of the lesion. However, he refused to undergo any surgery. The patient was then offered ablative modalities after explaining the risk of recurrence and malignant transformation associated with such procedures. The lesion was subsequently removed in a single sitting using a 2940 nm wavelength erbium-doped yttrium aluminum garnet (Er:YAG) laser at a fluence of 8.4 J/cm^2^, pulse width of 100 microseconds, and a laser spot size of 3 mm. The patient was prescribed topical mupirocin ointment post-procedure and was instructed to return for a follow-up appointment in two weeks. However, he was unable to follow up as he was residing at a considerable distance from our center.

## Discussion

Nevus sebaceous of Jadassohn is a congenital cutaneous hamartoma [10,11], which may only become evident as an individual grows older. It occurs most commonly over the scalp, followed by the face, preauricular area, and neck. Lesions may appear as hairless patches to slightly elevated plaques in childhood but progress to form verrucous to nodular lesions during adulthood [12].

The globules seen in nevus sebaceous give the lesions their classic verrucous or morbilliform appearance. Though globules are most commonly yellow, orange-brown globules have also been previously reported. Immature sebaceous glands lead to the formation of orange globules, while brown globules arranged in crateriform to cerebriform patterns represent the presence of hyperplastic, mature sebaceous glands [12]. On the other hand, warts, which are the closest differential diagnosis, show the presence of papillae with surrounding haloes as well as a brown-colored background and characteristic interruption of skin lines.

The diagnosis of nevus sebaceous can be confirmed by the characteristic histopathological features. Acanthosis and papillomatosis in the epidermis can be seen in virtually all cases. Spongiosis and parakeratosis are also commonly observed. The dermis shows perivascular and, less frequently, perifollicular mononuclear cell infiltrate. Similarly, skin appendage changes are also characteristic of nevus sebaceous. Increased sebaceous glands, which occur more often in lesions in adult patients, are seen in a vast majority of cases. An increase in ectopic apocrine glands in primitive hair follicles and a decrease in terminal hairs are also frequently observed. This propensity of nevus sebaceous to involve all components of the skin has led to it being called an organoid nevus [13].

Histopathological examination is also particularly important given the neoplasms associated with nevus sebaceous. Trichoblastoma and syringocystadenoma papilliferum are the most common benign tumors. Among malignant neoplasms associated with nevus sebaceous, basal cell carcinoma is the most common, with squamous cell, sebaceous, and apocrine carcinomas also being reported [10,11]. Benign tumors are seen in 13.6% of nevus sebaceous cases. On the other hand, malignant growth is seen in 1%-2.5% of cases [14]. The risk of malignant transformation increases with the age of the patient [10]. However, malignancies have also been sporadically reported in children [15].

Nevus sebaceous is primarily treated by a full-thickness surgical excision with a minimum of 2-3 mm margins through the epidermis, dermis, subcutaneous tissue, and underlying fat, with the excision being stopped at the level of the underlying fascia. Reconstruction with flaps may be required if the defect is too large [16]. Some authors even recommend prophylactic excision in healthy children because of their appearance on cosmetically sensitive areas and the resultant alopecia. Furthermore, an excision performed before enlargement of the lesion during puberty may lead to a less complicated surgery as well as a less noticeable scar. However, these advantages must be weighed against the risks of administering general anesthesia to children [17].

Other modalities such as electrocautery, fulguration, and curettage have also been used to treat nevus sebaceous. However, these methods may lead to incomplete clearance, recurrence, or masking of malignant changes [18,19]. Similarly, ablative lasers, which target the water inherent in the cells of the body as chromophores, such as 10600 nm carbon dioxide (CO_2_) and 2940 nm Er:YAG laser, have also shown unsatisfactory results [17]. A retrospective study in which 16 nevus sebaceous patients were treated with lasers showed a recurrence rate of 88%. However, half of these patients were satisfied even with the temporary or partial resolution of the lesions [20].

In our case, the patient did not consent to surgery despite being explained about the potential complications associated with the disease. He was also informed of the disadvantages of laser removal. However, the primary concern for the patient was cosmesis, which was why he instead opted for the removal of the lesion with an ablative Er:YAG laser.

## Conclusions

Nevus sebaceous is one of the lesions that can masquerade as a wart. However, a correct diagnosis is essential, given the associated neoplastic conditions. The predilection for the scalp with resultant alopecia and the tendency to increase in size from puberty are important clues toward diagnosis. Similarly, dermoscopy may help visualize characteristic globules, which helps in their identification. Histopathological examination is vital to rule out any associated malignant changes. Surgical full-thickness excision is the best treatment available for the condition. Prophylactic excision during childhood may be worth considering to reduce the incidence of malignancy and improve the cosmetic outcome of surgery. Laser ablation is a modality that may be offered when surgery is contraindicated or refused after explaining the risks associated with the procedure.
